# Novel insights into triple-negative breast cancer heterogeneity, prognosis, and treatment response based on matrix stiffness: a combined single-Cell and transcriptome analysis

**DOI:** 10.3389/fonc.2026.1821887

**Published:** 2026-05-07

**Authors:** Wenjie Shi, Haofeng Wang, Yingxin Guan, Yingzi Zhou, Jing Cui, Xiaojie Wang, Jinling Yu

**Affiliations:** 1 Department of Breast Surgery, Tongren Hospital, Shanghai Jiao Tong University School of Medicine, Shanghai, China; 2 Department of Pathology, Tongren Hospital, Shanghai Jiao Tong University School of Medicine, Shanghai, China

**Keywords:** Matrix stiffness, prognostic model, single-cell RNA sequencing, triple-negative breast cancer, tumor microenvironment

## Abstract

**Background:**

The heterogeneity of triple-negative breast cancer (TNBC) is closely related to its tumor microenvironment. Tumor matrix stiffness (MS) is a key physical factor regulating tumor progression. This study aimed to explore the impact of MS on the biological behaviors of TNBC and establish its clinical association with patient prognosis.

**Methods:**

In this study, by integrating the transcriptome data of TNBC patients from the TCGA and GEO databases and MS-related genes, initially, the cell MS score was calculated based on single-cell RNA-sequencing data to identify malignant cells. Signature genes were selected through differential expression screening, univariate/multivariate Cox regression, and machine-learning methods. An MS-based prognostic model was then constructed and validated in independent cohorts. Furthermore, a nomogram model was constructed to evaluate its prognostic prediction efficacy. Also, the role of the MS-score model in predicting the immune microenvironment and treatment response was assessed.

**Results:**

The single-cell atlas revealed that malignant epithelial cells with a high MS score had unique functional states and pathway characteristics. The prognostic model constructed based on MS-related genes could effectively predict the survival risk of patients in multiple cohorts. Multivariate analysis confirmed that the MS score was an independent prognostic factor. The nomogram constructed by incorporating clinical parameters demonstrated good calibration ability and clinical net benefit. At the treatment level, a high MS score was associated with immunotherapy resistance and decreased chemotherapy sensitivity.

**Conclusion:**

This study provides a new theoretical basis and translational tools for the prognostic assessment and precision treatment of TNBC.

## 1 Introduction

As an aggressive breast cancer subtype, triple-negative breast cancer (TNBC) lacks expression of estrogen receptor, progesterone receptor, and human epidermal growth factor receptor 2, and represents about 15-20% of breast cancer incidence (1, 2). This molecular characteristic renders it insensitive to targeted and endocrine therapies, and clinical treatment still mainly relies on chemotherapy (3). However, TNBC patients generally have a poor prognosis, a low overall survival rate, and their risk of death is significantly higher than that of other breast cancer subtypes (4, 5). Consequently, analyzing the pathological mechanisms of TNBC in depth, screening for reliable molecular biomarkers to improve clinical prognosis, and exploring novel therapeutic targets are of significant importance.

The high heterogeneity of the tumor microenvironment (TME) has become a key determinant of treatment resistance and prognostic differences in TNBC (6). The TME comprises both cellular and non-cellular components. The cellular components include tumor cells, cancer-associated fibroblasts, and various immune cells, while the non-cellular components are primarily composed of the extracellular matrix (7). These two components interact continuously to jointly regulate tumor evolution and treatment response, which is mainly reflected in three core aspects, including immune infiltration status, metabolic adaptation, and spatial tissue structure (8–10). This profoundly affects the biological behavior, immunotherapy sensitivity, and clinical outcomes of TNBC.

Among the many physical characteristics of the TME, matrix stiffness (MS), as a key mechanical factor, is a core manifestation and driving force of its heterogeneity (11). MS is non-uniformly distributed within the tumor, and its spatial variation directly results from the local deposition, cross-linking, and remodeling of ECM components such as collagen (12). Relevant studies have indicated that increased MS can induce a stem-like phenotype in cancer cells via Mechan transduction signaling pathways, enhancing their tumorigenicity and treatment resistance (13, 14). At the same time, MS can promote the polarization of macrophages into the protumorigenic M2 phenotype, thus establishing an immunosuppressive microenvironment that accelerates tumor progression and weakens the efficacy of immunotherapy (15). In addition, at the metabolic level, higher MS can drive metabolic reprogramming in breast cancer cells, exacerbating treatment resistance (16). Therefore, systematically constructing the molecular characteristics related to MS and clarifying its role in the prognostic assessment of TNBC are of great significance for revealing the pathogenesis of this disease, improving risk stratification, and developing new treatment strategies.

This study revealed the MS-regulated molecular characteristics through single-cell transcriptomic analysis, by integrating The Cancer Genome Atlas (TCGA) and Gene Expression Omnibus (GEO) transcriptomes with MS-related gene sets. Subsequently, using machine learning, prognostic feature genes were identified, which were then utilized to construct a prognostic prediction model. Validation of the model’s independent prognostic value was performed using both univariate and multivariate Cox regression analyses. Furthermore, nomograms and calibration curves were constructed and plotted for the assessment of the model’s predictive efficacy. Genomic analysis was combined to confirm its stability, and its ability to predict immunotherapy response and drug sensitivity was validated in an independent cohort. Finally, the expression characteristics of the characteristic genes were located to reveal their potential biological functions. This study provides new clues for clarifying the molecular mechanism by which MS regulates the progression of TNBC, and offers potential molecular tools for prognostic assessment.

## 2 Materials and methods

### 2.1 Data sources

The bulk RNA-sequencing data of TNBC were obtained from the GEO and TCGA databases respectively (17, 18). The TNBC samples from the TCGA clinical data were used as the training set, including a total of 124 TNBC samples and 113 normal breast samples. GSE21653 (82 TNBC samples) and GSE163882 (90 TNBC samples) were used as the validation sets. The single-cell RNA-sequencing data were retrieved from the GEO database (GSE176078, GSE161529), including a total of 13 normal breast samples and 14 TNBC samples. The datasets GSE163882 (90 TNBC samples) and GSE103668 (21 TNBC samples) were the immunotherapy data of TNBC. The MS-related gene set was obtained by integrating relevant pathways in MSigDB and literatures (19). Entries with fewer than 20 genes or containing non-coding RNAs were excluded, and finally 1,418 genes were obtained.

### 2.2 Processing of single-cell data and identification of tumor cells

The Seurat package was employed for processing the single-cell RNA-sequencing data (20) (Version 5.2.1, https://cran.r-project.org/web/packages/Seurat/index.html). First, a Seurat object was created and quality control filtering was performed. Cells with the number of genes ranging from 500 to 7000, total unique molecular identifiers from 200 to 50,000, and the proportion of mitochondrial genes less than 20% were retained. Subsequently, the FindClusters function was used for cell clustering (resolution = 0.2), and highly variable genes were identified through FindVariableFeatures (logfc.threshold = 0.25, min.pct = 0.1, only.pos = TRUE). After batch effect correction using the harmonyR package, cell types were identified through manual annotation. To identify malignant cells, based on the annotation results, the run function of the inferCNV software package (Version 1.22.0) was further utilized to perform copy-number variation (CNV) analysis on relevant cells (21). Normal cell clusters were used as references to detect amplifications and deletions of chromosome regions in tumor cells, and the CNV score of each cluster was calculated to identify malignant cells.

### 2.3 Construction of the MS scoring model and cell trajectory analysis

The MS score of each cell was calculated using the cal_CRDscore function from the CRDscore package (22) (Version 0.1.0, https://github.com/leihe2021/CRDscorel). Cells were then divided into high- and low-score subgroups based on the optimal cutoff value of the MS score. The Wilcoxon rank-sum test was employed to evaluate the differences in MS scores of malignant cells under different conditions, and Gene Set Variation analysis (23) (GSVA, Version 2.2.0, https://www.bioconductor.org/packages/release/bioc/html/GSVA.html) was utilized to analyze the enrichment degree of Kyoto Encyclopedia of Genes and Genomes (KEGG) pathways between high- and low-MS-score malignant cells. To explore the evolutionary trajectory of malignant cells, Monocle was further applied to construct a pseudotime trajectory that included normal cells and high- and low-MS-score malignant cells (24). For interrogating the differentially expressed genes between high- and low-MS-score malignant cells, the clusterProfiler package was applied to carry out KEGG pathway enrichment analysis (25).

### 2.4 Characterization of the tumor immune microenvironment and cell-to-cell communication analysis

Based on the MS scores, samples were classified into high- and low-MS-score risk groups. A quantitative comparison was made of the proportions of malignant cells, immune cells, and other cell types between the two groups. The differences in the expression levels of tumor-associated antigens (TAA) and major histocompatibility complex (MHC) molecules between the two groups were evaluated. Additionally, the ssGSEA algorithm within GSVA was employed to quantitatively assess the enrichment of immunogenic cell-death pathways and T-cell-related functions. Regarding cell-to-cell communication, using the CellphoneDB approach in the CellChat package (26) (Version 1.6.1, https://github.com/jinworks/CellChat), communication probabilities were calculated via its built-in ligand-receptor database, followed by statistical tests. Significant ligand-receptor pairs were screened to compare the interaction differences between the two groups. Simultaneously, by integrating the scaper tool (27) (Version 0.2.0, https://search.r-project.org/CRAN/refmans/scaper/html/scapeForSeurat.html) with the CytoSig and Reactome databases, the transcriptional activities of cytokine-related signaling pathways in each group were quantitatively evaluated.

### 2.5 Construction of the MS score prognostic model

Based on the TCGA data, the DESeq2 package (sion 1.47.5, https://bioconductor.org/packages/release/bioc/html/DESeq2.html) was used to identify differentially expressed genes (DEGs) between TNBC and normal tissues (*P* < 0.05 and |log2 Fold Change (FC)| > 1). The clusterProfiler package was then utilized to conduct GO and KEGG enrichment analyses on the intersecting genes between the DEGs and the known MS-related genes. To construct the MS score prognostic model, prognostic-related genes were first screened from the intersecting genes through univariate Cox regression. Subsequently, 101 combinations of 10 machine-learning algorithms were employed for modeling. The optimal model with the highest average C-index and a C-index greater than 0.6 in two independent datasets was selected, and the genes included in this model were defined as characteristic genes. Finally, the MS score of each patient was calculated based on this model. According to the optimal cut-off value of the MS score, the sample patients were divided into high- and low-MS-risk groups. The prognostic prediction performance of the model was evaluated by Kaplan-Meier curves and ROC curves in the training set and validation sets. Meanwhile, the distribution of MS scores and the distribution of survival status were presented to intuitively demonstrate the performance of the model.

### 2.6 Determination of the prognostic value of the MS score model

First, by integrating the clinical data of the training set and validation sets, the C-index of clinical factors in each dataset was calculated in combination with the MS score. Subsequently, through univariate and multivariate Cox regression analyses of the training set, the value of the MS risk score as an independent prognostic factor was evaluated. To verify the efficacy of the model, nomograms and calibration curves were further constructed using the rms package (28) (Version 6.8.2, https://cran.r-project.org/web/packages/rms/index.html), and decision-curve analysis was performed using the rmda package (29) (Version 1.6, https://github.com/mdbrown/rmda/releases). Thirteen prognostic signatures related to triple-negative breast cancer published in the past decade were retrieved and included. By comparing the C-indices in the TCGA training set and the validation sets of GSE58812 and GSE21653, the prognostic prediction abilities of the MS score and these reported signatures were evaluated.

### 2.7 Evaluating the prognostic value of the MS score model via integrated genomic analysis

The GISTIC2.0 tool (30) (https://cloud.genepattern.org/gp/pages/index.jsf) was used to first compare differences in recurrent copy number alterations between the high- and low-MS-risk groups. The maftools package (31) (Version 2.24.0, https://www.bioconductor.org/packages/release/bioc/html/maftools.html) was employed to analyze the gene mutation spectra and tumor mutational burden (TMB) in the two groups. Furthermore, the high- and low- MS-score groups were each divided into four subgroups, namely H_TMB_High, H_TMB_Low, L_TMB_High, and L_TMB_Low, according to the median of TMB. Kaplan-Meier survival analysis was then conducted to evaluate the prognostic differences among these subgroups.

### 2.8 Role of the MS score model in predicting the immune microenvironment and treatment response

A combination of the IOBR and GSVA packages, together with the ssGSEA algorithm, was employed for a comprehensive assessment of the tumor immune microenvironment. The differences in immune cell infiltration and the expression of immunomodulatory genes between the high- and low- MS-score groups were compared. To further assess the predictive potential of the MS score, the associations between the MS score and multiple immunotherapy biomarkers were analyzed, and its predictive efficacy for the response to PD-1/PD-L1 inhibitors was validated in independent immunotherapy cohorts (GSE163882, GSE103668). Meanwhile, the oncoPredict package (32) (Version 2.24.0, https://github.com/HuangLabUMN/oncoPredict) was applied to calculate the IC50 values of common chemotherapeutic and targeted drugs. Subsequently, drug sensitivity was compared between the high- and low-MS groups.

### 2.9 Analysis of expression characteristics and biological associations of characteristic genes

A comparative analysis was conducted of the expression differences of characteristic genes, both between tumor and normal tissues and across different cell types. The differentiation trajectories of characteristic genes in malignant cells were analyzed, and the correlation between the MS score of tumor cells and the expression of characteristic genes was evaluated. To explore potential biological connections, the correlations between each characteristic gene, the MS score, and the expression of STAT3 were further analyzed.

### 2.10 Reverse transcription-quantitative polymerase chain reaction detection

Total RNA extraction from TNBC tissue samples was performed with a dedicated kit for paraffin-embedded sections (TIANGEN Biotech, Beijing, China). After undergoing processes such as dewaxing, digestion, centrifugal purification, on-column DNase I digestion, and elution through rinsing, the RNA was obtained. Its concentration was then measured. Subsequently, First-Strand cDNA Synthesis SuperMix for Qpcr (TransGen Biotech, Beijing, China) was utilized for reverse transcription to generate cDNA. Finally, using this cDNA as a template, fluorescence-based quantitative PCR amplification was carried out with PerfectStart^®^ Green qPCR SuperMix (TransGen Biotech) and the specific primers listed in **Supplementary Table 1**. Additionally, a melting curve analysis was performed.This study was approved by the Ethics Committee of Tongren Hospital,Shanghai Jiao Tong University School of Medicine (Formerly known as Shanghai Changning District Maternal and Child Health-Care Hospital)(Approval No.CNFBLLKTY-IEC-2025-008).Written informed consent was obtained from all patients, or the requirement for informed consent was waived due to the retrospective nature of the study using archival tissue samples.

### 2.11 Statistical analysis

Statistical analysis was performed using R software (version 4.3.1). Comparisons of continuous variables between groups were conducted using the Wilcoxon rank-sum test. Correlation analysis was performed using Pearson or Spearman methods. Comparisons among multiple groups were conducted using ANOVA test. A two-sided *P* < 0.05 was considered statistically significant. Data visualization was performed using GraphPad Prism 5 (GraphPad Software, San Diego, CA), *P* < 0.05 and *P* < 0.01 used as thresholds for significant.

## 3 Results

### 3.1 Unraveling the heterogeneity of TNBC cells through single-cell transcriptomics

**Figure 1:**
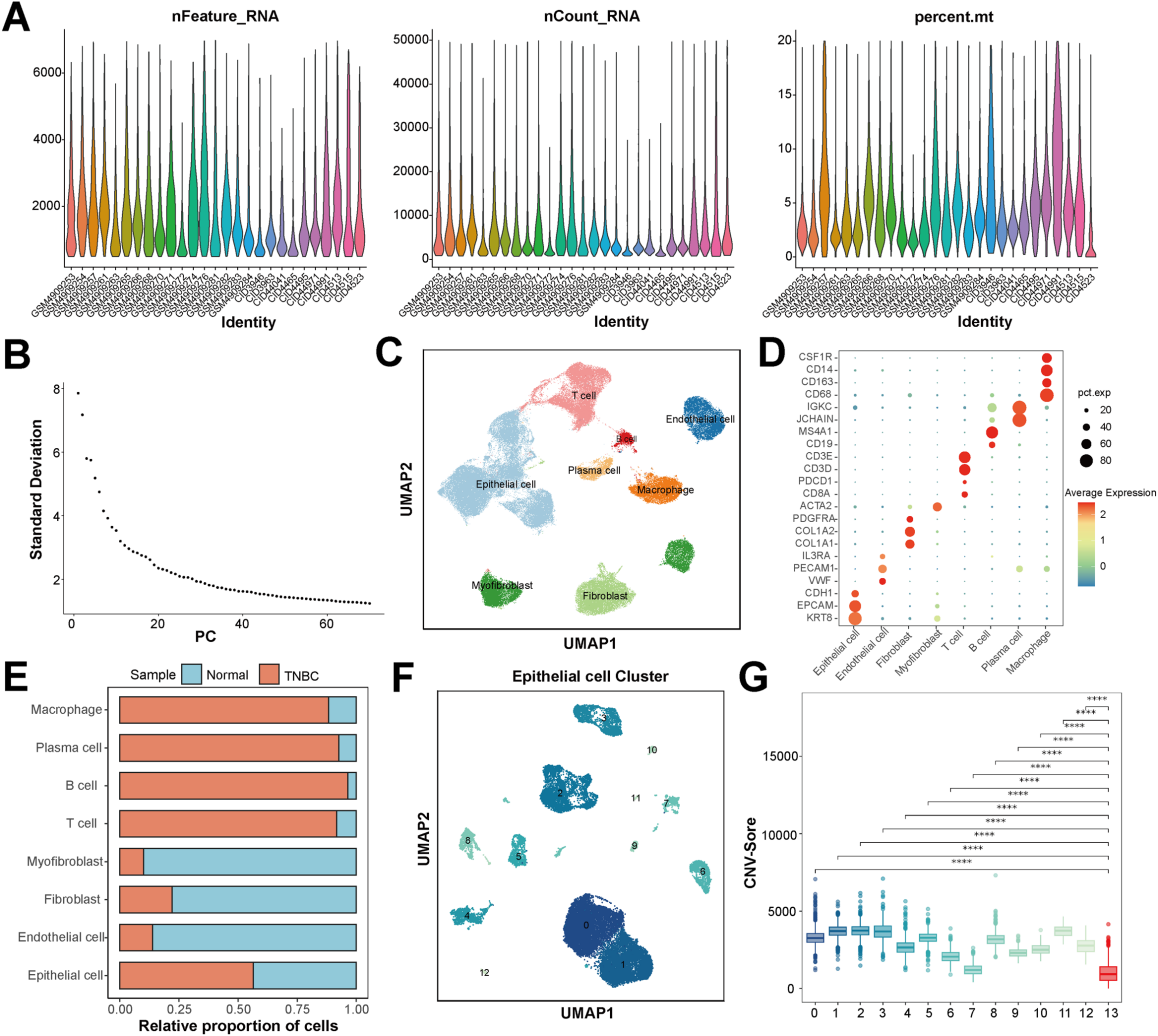
Single-cell sequencing reveals the cell atlas of triple-negative breast cancer (TNBC). **(A)** Violin plot of single-cell quality control analysis, showing the distribution of the number of unique genes detected per cell (nFeature_RNA), the total number of transcripts (nCount_RNA), and the percentage of mitochondrial gene expression (percent.mt). **(B)** Principal component analysis used to determine the dimensionality of dimensionality reduction. **(C)** UMAP plot of cell-type annotation based on known marker genes, with colors representing cell types. **(D)** Bubble plot of the expression of key marker genes in each cell type. The shade of the color represents the average expression level of the gene in the corresponding cell type, and the size of the circle represents the proportion of cells expressing the gene. **(E)** Proportion distribution of each cell type in the normal group and the TNBC group. **(F)** Clustering plot of UMAP sub-populations of epithelial cells sorted from tumor samples. **(G)** Copy-number variation (CNV) scores of each epithelial cell sub-population. * p < 0.05, ** p < 0.01, *** p < 0.001, **** p < 0.0001, ns = not significant.

To analyze the heterogeneity of sample cells and identify their malignant components, quality control was first performed on the single-cell data. As shown in **Figure 1A**, after quality control, the distributions of the number of genes, total number of transcripts, and the proportion of mitochondrial genes in the retained cells of each sample were more concentrated and reasonable, and the data quality was significantly improved.

The data was further normalized and scaled, and according to the elbow plot, the first 40 principal components that retained most of the variation information were determined for subsequent dimensionality-reduction analysis (**Figure 1B**). Based on the dimensionality-reduction results of the principal components, through cell clustering and UMAP visualization, and combined with the marker genes in the literature and the Cell Marker 2.0 database, we identified eight major cell types, namely epithelial cells, endothelial cells, fibroblasts, myofibroblasts, T cells, B cells, plasma cells, and macrophages (**Figures 1C, D**).

By comparing the cell composition of TNBC samples and normal samples, significant differences were found in the distribution of cell subsets. The proportions of B cells, T cells, plasma cells, macrophages, and epithelial cells in TNBC samples all exceeded 50% (**Figure 1E**). Given that TNBC is an epithelial-derived malignant tumor, the epithelial cells in tumor samples were reclustered, and the epithelial cells were divided into 13 cell clusters (**Figure 1F**). To assess the malignancy of epithelial cells, with the epithelial cell clusters in normal samples as a reference, the CNVs were analyzed. As shown in **Figure 1G**, all epithelial cell clusters from tumors exhibited significantly elevated CNV scores compared to the normal reference group, validating the definitive malignant nature of the sampled epithelial cells.

### 3.2 MS characteristics influence the functional states of malignant epithelial cells in TNBC

**Figure 2:**
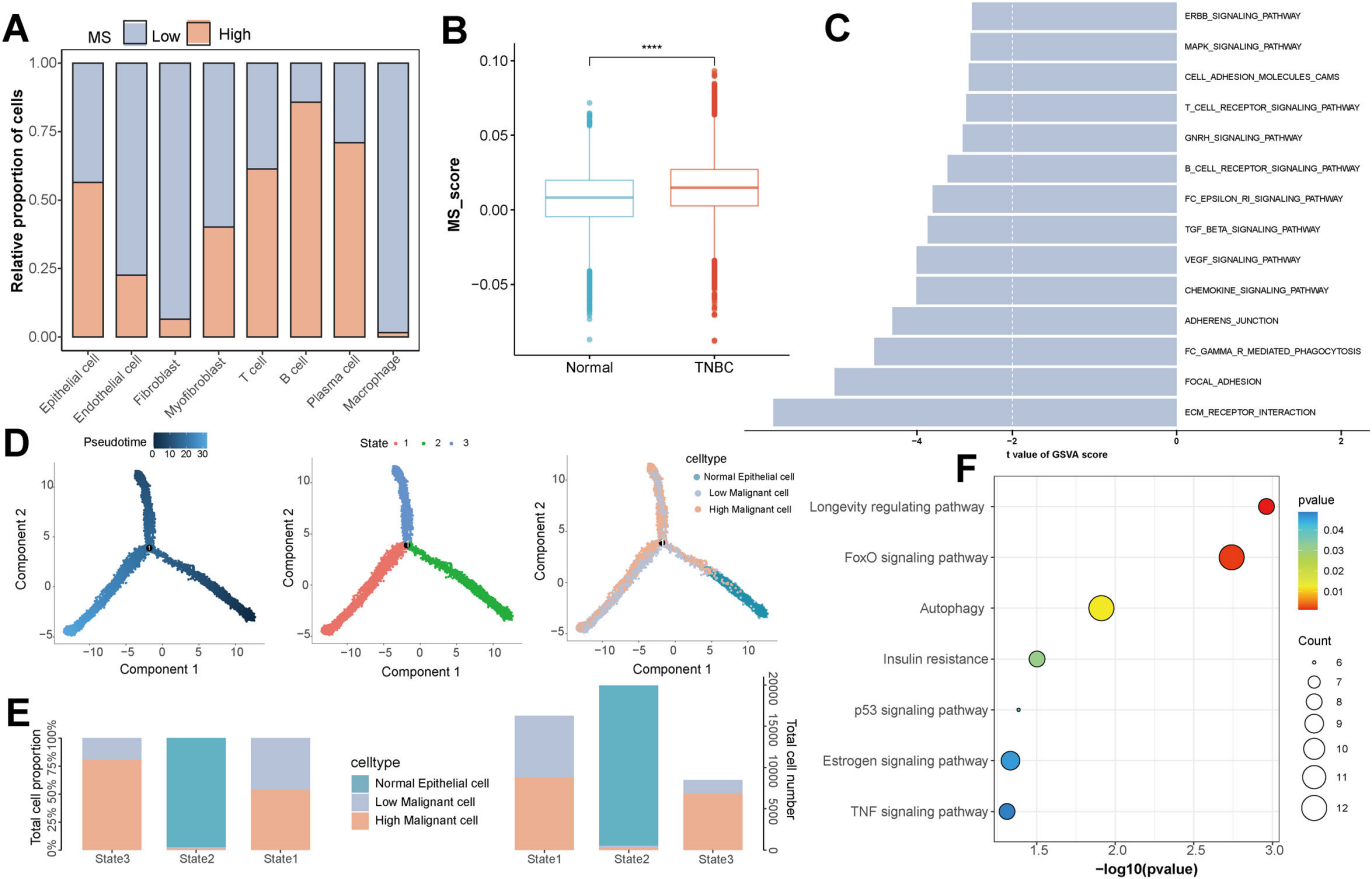
Characterization and functional analysis of MS-related signals at the single-cell level in TNBC. **(A)** The composition ratio of the number of cells in the high- and low-MS score groups in each cell type. **(B)** Comparison of MS scores in epithelial cells from normal breast tissues and TNBC tissues. **(C)** In the identified malignant epithelial cells, KEGG pathway enrichment analysis based on GSVA was performed for the high-and low-MS score groups. **(D)** Pseudotemporal analysis: From left to right are the trajectory distributions of epithelial cells colored by pseudotemporal values, cell states, and MS score groups. **(E)** The number and percentage of cells in the high- and low-MS score groups in each cell state defined along the pseudotemporal trajectory. **(F)** KEGG pathway enrichment analysis of differentially expressed genes between the high- and low-MS score groups.

To clarify the role of MS in the functional heterogeneity of tumor cells, we calculated the MS score for each cell based on 1,418 MS-related genes. Using the median MS score of tumor cells as the boundary, the cells were dichotomized into high- and low-score groups. Analyzing the proportion of high- and low-score groups in each cell type revealed that B cells, T cells, plasma cells, and epithelial cells showed a markedly higher proportion of high-MS-score cells compared to other cell types (**Figure 2A**). Moreover, in contrast to normal samples, epithelial cells in TNBC samples displayed significantly higher MS scores (**Figure 2B**).

To reveal the molecular characteristics of malignant epithelial cells, we compared the pathway enrichment differences between malignant epithelial cells with high and low MS scores. As depicted in **Figure 2C**, in malignant epithelial cells with high MS scores, multiple pathways, including ERBB, MAPK, T/B cell receptor signaling pathways, focal adhesion, and extracellular matrix-receptor interaction, exhibited a downward-trending pattern. To analyze the state evolution of malignant epithelial cells in the pseudotime dimension, trajectory analysis was conducted. The cells differentiated into three states along the pseudotime axis. Normal epithelial cells were predominantly enriched in State 2 at the start of the trajectory, while malignant cells with high MS scores were significantly concentrated in State 3 (**Figures 2D, E**).

To further uncover the regulatory disparities between the high- and low-MS-score groups of malignant epithelial cells, performing an enrichment analysis on the DEGs was conducted. As shown in **Figure 2F**, the FoxO signaling, autophagy, p53 signaling, and TNF signaling pathways were significantly enriched. Collectively, these results demonstrated that the MS score could effectively distinguish different functional states of tumor epithelial cells, suggesting that MS represented a crucial dimension in regulating the malignant progression of TNBC.

### 3.3 High MS score characterizes immune microenvironment remodeling and abnormal cell communication

**Figure 3:**
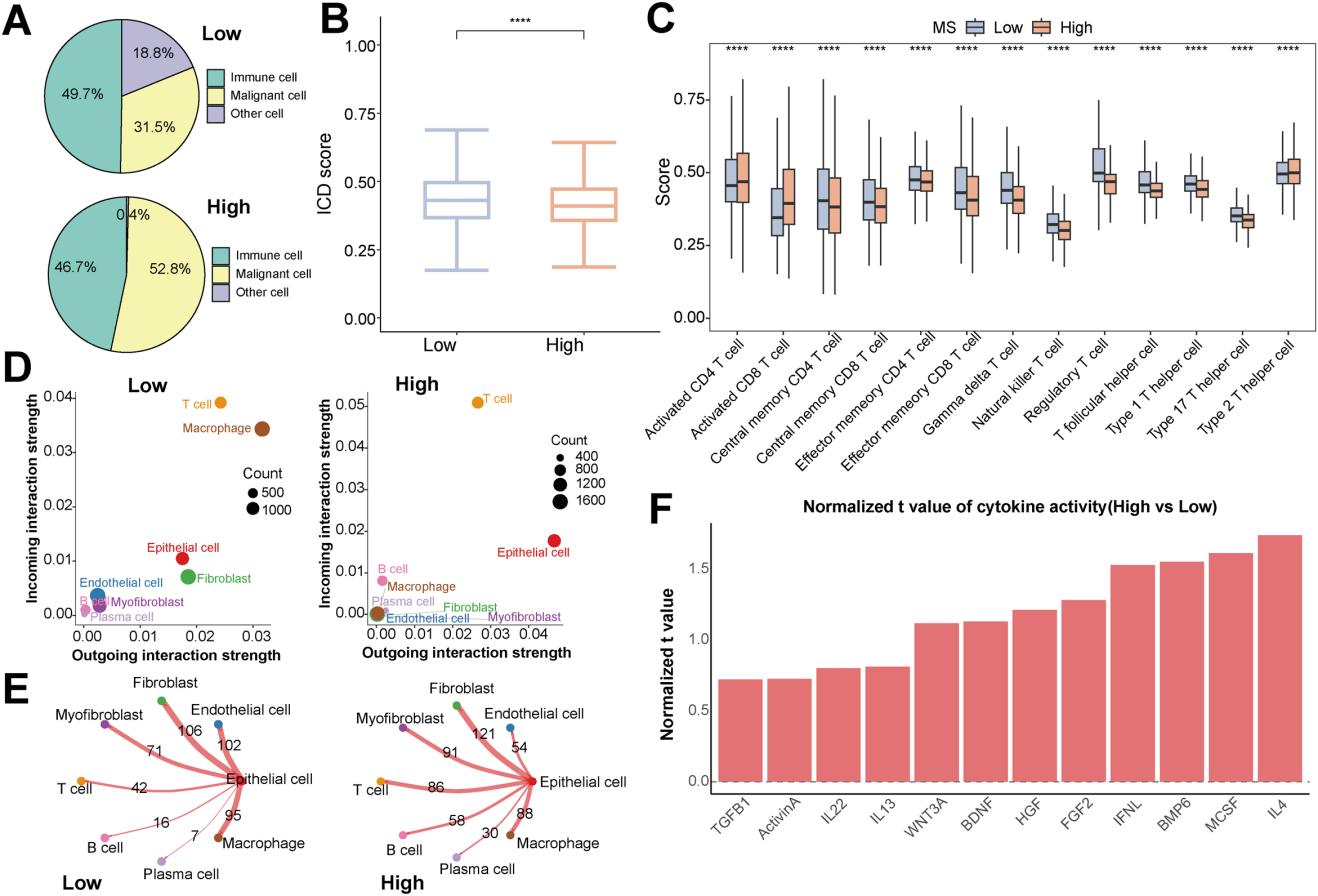
Impact of MS-related signals on the composition and interactions of cells in the tumor microenvironment. **(A)** Distribution of the proportions of malignant epithelial cells, immune cells, and other cell types in samples of the high- and low-MS score groups. **(B)** Comparison of the activities of the immunogenic cell death (ICD) pathway between the high- and low-MS score groups. **(C)** Differences in the scores of 13 key T-cell-related functions between the high- and low-MS score groups. **(D)** Spatial distribution of cells in the high- and low-MS score groups based on high-dimensional data dimensionality reduction. **(E)** Comparison of the predicted number of cell-cell communications between epithelial cells and all other cell types in the high- and low-MS score groups. **(F)** Ranking of cytokines with significantly upregulated cytokine activity scores in the high-MS score group compared to the low-MS score group. * p < 0.05, ** p < 0.01, *** p < 0.001, **** p < 0.0001, ns = not significant.

To clarify the interplay between the MS score and the tumor immune microenvironment, we first analyzed the differences in cell composition between groups with different MS scores. As depicted in **Figure 3A**, an increase in the proportion of malignant cells in the high-MS group was accompanied by a corresponding decrease in immune and other stromal cells. Correspondingly, the overall expression of TAA and MHC molecules was also significantly downregulated in the high-MS group (**Supplementary Figure 1A**). Functional analysis indicated notably lower immunogenic cell death scores in the high-MS group and extensive disparities in T-cell-related functions (**Figures 3B, C**).

Cell-communication network analysis revealed that T cells and macrophages were the communication hubs in the low-MS group, while in the high-MS group, epithelial cells and T cells were dominant (**Figure 3D**). Further analysis of epithelial-T cell interactions revealed that the receptor-ligand pairs MIF−(CD74 + CD44), MIF−(CD74 + CXCR4), and MDK−NCL were expressed at significantly higher levels compared to other pathways, especially in the high-MS group (**Supplementary Figure 1B**).

Notably, the shift in the communication pattern was accompanied by a significant change in connection strength. In contrast to the generally enhanced interactions with other cell types, epithelial cells in the high-MS group exhibited diminished communication with endothelial cells and macrophages (**Figure 3E**). Additionally, 12 cytokines with significantly upregulated activities were identified in the tumor epithelial cells of the high-MS group (**Figure 3F**). These results provide evidence for the close association of a high-MS score with the immune microenvironment, along with accompanying substantial alterations in the cell-communication network.

### 3.4 Construction and validation of a TNBC prognostic model Based on MS-related genes

**Figure 4:**
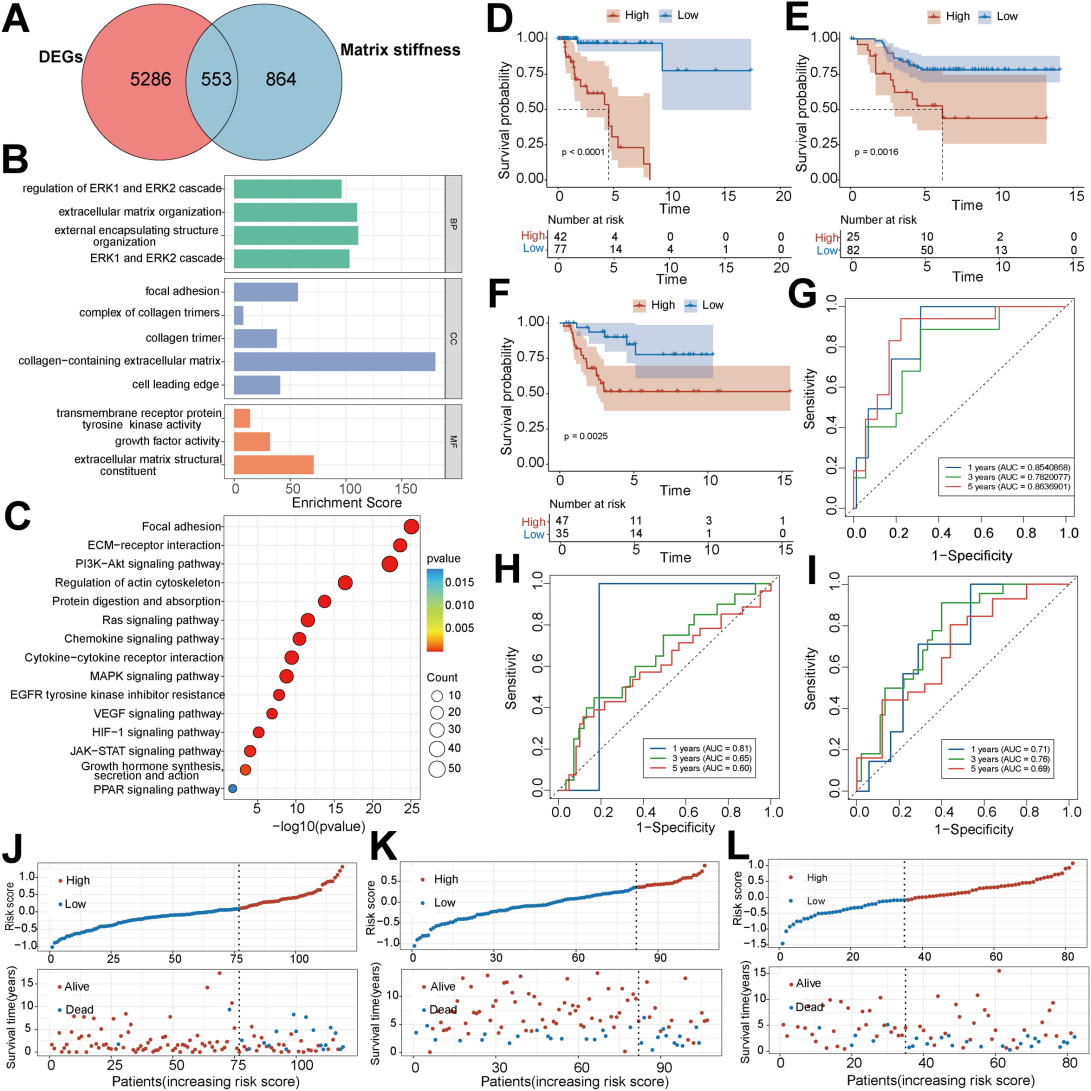
Construction and multi-dataset validation of the prognostic model for MS signals. **(A)** Venn diagram of the intersection between MS-related genes and transcriptome differentially expressed genes. **(B)** Bar plot of Gene Ontology (GO) functional enrichment analysis of the intersecting genes. **(C)** Bubble plot of KEGG pathway enrichment analysis of the intersecting genes. **(D–F)** In the TCGA cohort **(D)**, GSE58812 dataset **(E)** and GSE21653 dataset **(F)** Kaplan-Meier survival curves were used to evaluate the predictive ability of the MS prognostic model for patient survival outcomes. G-I, In the TCGA cohort **(G)** GSE58812 dataset **(H)** and GSE21653 dataset **(I)** 1-year, 3-year, and 5-year receiver operating characteristic (ROC) curves were plotted respectively to evaluate the predictive performance of the MS prognostic model at different time points. **(J–L)**, Distribution of MS scores and survival status of patients were shown in the TCGA cohort **(J)**, GSE58812 dataset **(K)**, and GSE21653 dataset **(L)** respectively.

To explore the biological functions and prognostic values of MS-related genes in TNBC, we first analyzed the differential expression in TCGA transcriptome data and obtained 5,839 DEGs. The intersection of the DEGs and the MS-related gene set contained 553 genes (**Figure 4A**). The results of functional enrichment analysis highlighted the predominant involvement of these genes in ERK signal regulation and extracellular matrix-associated biological processes. They aggregated in the focal adhesion cell structure and were associated with the functions of transmembrane receptor tyrosine kinase and growth factor activity (**Figure 4B**). KEGG pathway analysis further pinpointed the enriched pathways of these genes — prominently including the PI3K-Akt and MAPK signaling pathways, along with tumor microenvironment-related pathways like ECM-receptor and cytokine-cytokine receptor interactions (**Figure 4C**).

After intersecting the screened prognosis-associated genes with the validation set, 14 characteristic genes were ultimately obtained for constructing the prognostic signature. Subsequently, patients were stratified into high- and low-risk groups based on the median MS score, following calculation of each patient’s MS score from the optimal model. Kaplan-Meier analysis revealed a markedly poorer prognosis for high-risk group patients, both in the training and validation sets (**Figures 4D–F**). The time-dependent ROC curves indicated that the MS score model performed well in discrimination at 1-, 3-, and 5-year time points, with corresponding AUC values all above 0.6 (**Figures 4G–I**). In addition, in three independent datasets, a high-risk score was significantly associated with a higher proportion of deaths, further validating the stability of this prognostic model (**Figures 4J–L**).

### 3.5 Independent prognostic value of MS score and construction of clinical prediction model

**Figure 5:**
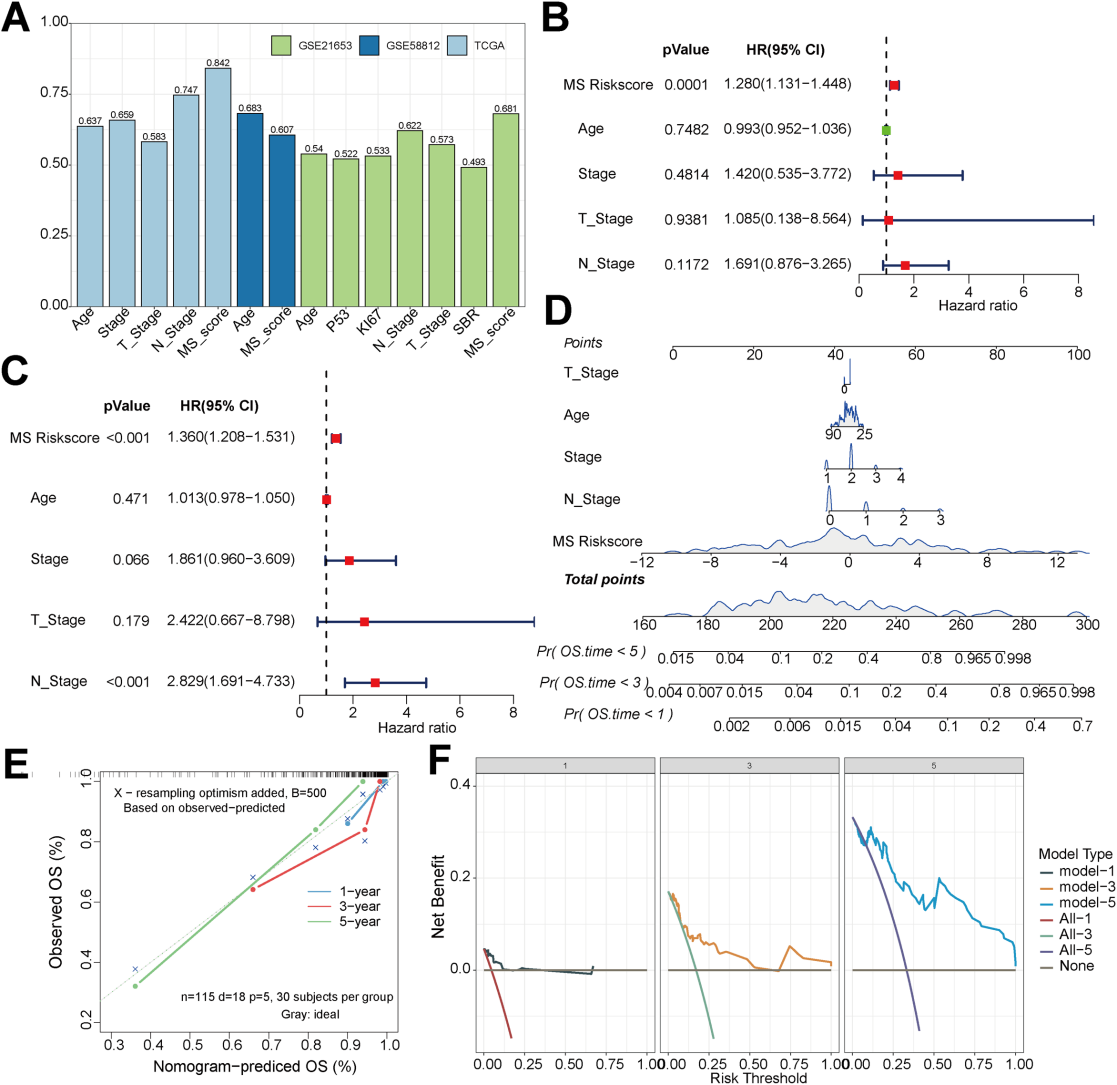
Construction of the MS prognostic nomogram and assessment of its clinical utility. **(A)** Comparison of the concordance index (C-index) of the MS scoring model in the training set and the validation set. **(B)** Univariate Cox regression analysis based on clinical variables and the MS score in the TCGA training-set cohort. **(C)** Multivariate Cox regression analysis in the TCGA training-set cohort. **(D)** Individualized survival-prediction nomogram constructed by integrating the independent prognostic factors obtained from the multivariate Cox regression analysis. **(E)** Calibration curves for the consistency between the 1-year, 3-year, and 5-year overall survival rates predicted by the nomogram model and the actually observed survival rates. **(F)** Decision curve analysis (DCA) of the nomogram model at the 1-year, 3-year, and 5-year time points. The red, green, and purple curves represent the strategy of all patients experience the clinical outcome, and the gray curve represents the strategy of no patient experiences the clinical outcome.

To evaluate the clinical prognostic value of the MS score, the C-indices of various clinical factors in the training set and the validation set were calculated and compared. As shown in **Figure 5A**, the MS score demonstrated better predictive performance than traditional clinical indicators in multiple independent datasets. The MS score was subsequently validated as an independent prognostic predictor for TNBC patients through both univariate and multivariate Cox regression analyses (**Figures 5B, C**). A prognostic nomogram incorporating the MS score and other key clinical parameters was further constructed (**Figure 5D**). By summing up the scores of each variable, the overall survival probabilities of patients at 1-, 3-, and 5-year could be predicted respectively. Among them, the MS score and N-stage contributed the most significantly to the total score. Further validation showed that the predicted survival rates in the calibration curve were highly consistent with the actual observed values, and the decision curve analysis confirmed that this model had significant clinical net benefits within a wide range of thresholds. The calibration curve and the decision curve together demonstrated that this nomogram had good predictive accuracy and clinical applicability (**Figures 5E, F**).

### 3.6 Predictive efficacy and synergistic effects of MS score

**Figure 6:**
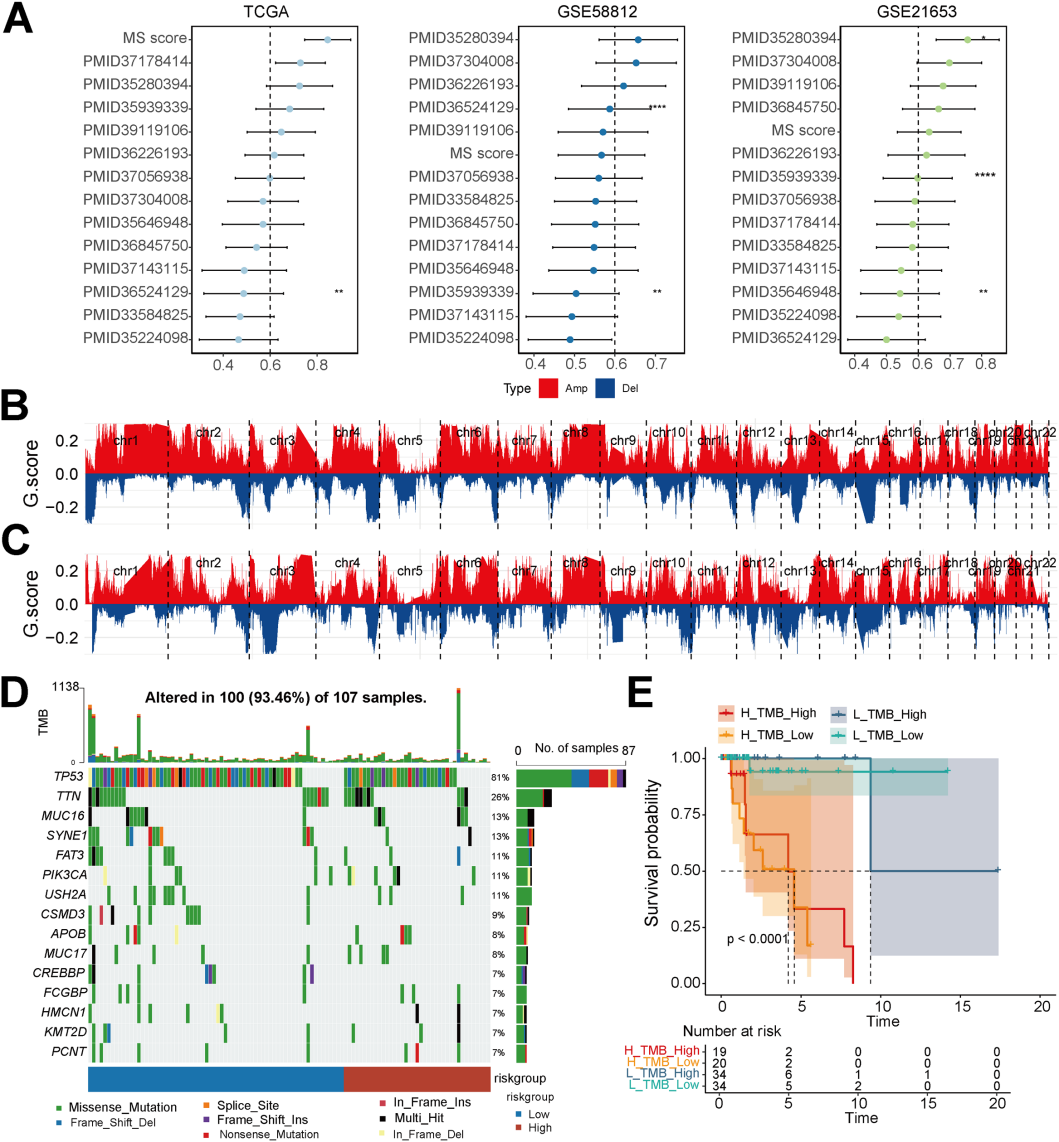
Comparative advantages of the MS prognostic model and its relationship with genomic features. **(A)** Comparative analysis of the predictive performance of the MS-score prognostic model with classical prognostic gene signatures published in the past decade in three independent datasets, TCGA, GSE58812, and GSE21653. **(B)** Overall landscape of genomic copy-number variation (CNV) in TNBC samples with a high MS score. **(C)** Overall landscape of genomic CNV in TNBC samples with a low MS score. **(D)** Mutation profiles of genes with high-frequency mutations in the high- and low-MS score groups. **(E)** Kaplan-Meier survival analysis with combined grouping of MS score and TMB to evaluate survival differences among four groups of patients (H_TMB_High, H_TMB_Low, L_TMB_High, L_TMB_Low).

To benchmark the model against existing standards, the MS score was compared with multiple previously published TNBC prognostic signatures. As shown in **Figure 6A**, the MS score exhibited the best predictive performance in multiple datasets, with a significantly higher C-index than other models. At the genomic level, copy-number variation analysis revealed that the high-MS score group had a more significant variation frequency on chromosomes 4 and 14 (**Figures 6B, C**). Analysis of the gene mutation spectrum showed that both groups were dominated by missense mutations, with a high-frequency mutation in TP53 (**Figure 6D**).

To explore the combined prognostic value, we further combined the MS score with TMB for subgroup analysis. Kaplan-Meier survival analysis revealed significant overall survival disparities across the four combined subgroups. Patients in the high-MS score combined with high-TMB (H_MS_High_TMB) group had the worst prognosis, indicating a synergistic effect between the MS score and TMB in TNBC prognostic stratification (**Figure 6E**). Collectively, these results confirmed that the MS score was a reliable tool for prognostic assessment, and its combination with genomic variation characteristics could provide more refined risk stratification.

### 3.7 Association of MS score with immune microenvironment and treatment response

**Figure 7:**
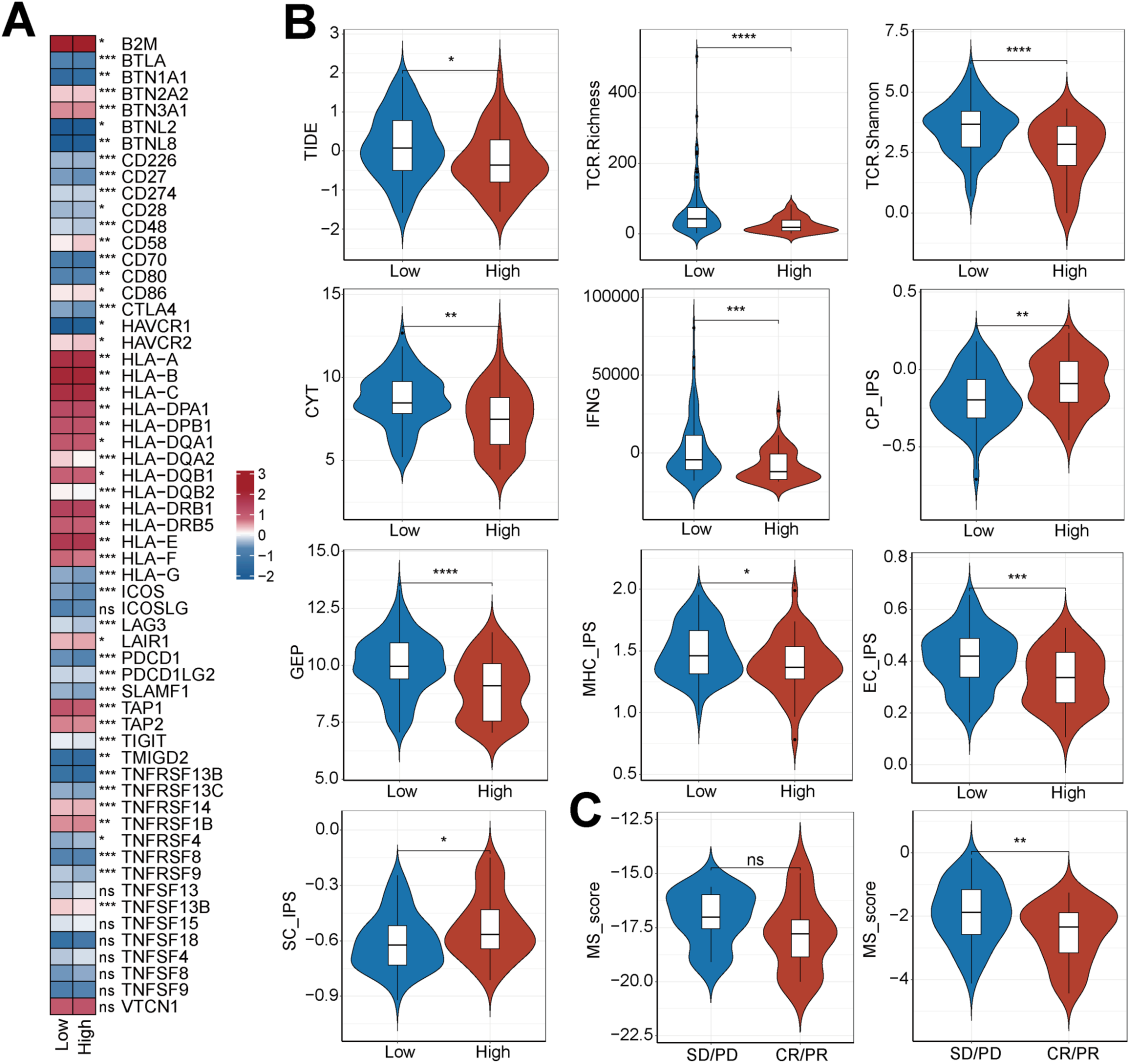
Association between MS signals and immunotherapy characteristics and efficacy prediction. **(A)** Correlation heatmap between high- and low-MS score groups and the expression levels of key immunomodulatory molecules. **(B)** Comparative analysis of the expression levels of established clinical predictors of immunotherapy in the high- and low-MS score groups. **(C)** The MS-score model was directly applied to two independent immunotherapy cohorts (GSE103668 and GSE163882) to compare the distribution differences of MS scores between the treatment-responsive and non-responsive groups.

To investigate the relationship linking the MS score to the tumor immune microenvironment and therapeutic outcome, we initially examined the immune cell infiltration profile. The results of ssGSEA showed that the infiltration levels of most immune cells (activated T cells, B cells, natural killer cells, and myeloid cells) were positively correlated with a high-MS score (**Supplementary Figure 2A**). However, the expression of immune-function-related genes presented a differential pattern. In contrast to antigen-presenting genes (HLA-A/B/C, TAP1/2), which showed positive correlation with the MS score, the expression of molecules involved in cell adhesion (CD226, SLAMF1) and immune checkpoint pathways (PDCD1, CD274) correlated negatively with the MS score. (**Figure 7A**).

Subsequently, to evaluate the value of the MS score in predicting immunotherapy, we analyzed its association with multiple predictive factors. As shown in **Figure 7B**, indicators reflecting anti-tumor immune activity (such as CYT, GEP, TCR diversity, IFNG) were significantly decreased in the high-MS-score group, while indicators related to immunosuppression and stromal function (such as CP-IPS, SC-IPS) were significantly increased in this group. In an independent immunotherapy cohort, the MS score of non-responder patients was significantly higher than that of responder patients (**Figure 7C**). Drug sensitivity analysis further indicated that the low-MS score group was significantly more sensitive to a variety of chemotherapeutic drugs (such as paclitaxel, gemcitabine, cisplatin) and targeted drugs (such as PARP inhibitors, PI3Kα inhibitors) (**Supplementary Figure 2B**). These results suggested that the MS score had the potential to be a dual biomarker for predicting tumor immune status and treatment response.

### 3.8 Role of characteristic genes in the malignant progression of TNBC

**Figure 8:**
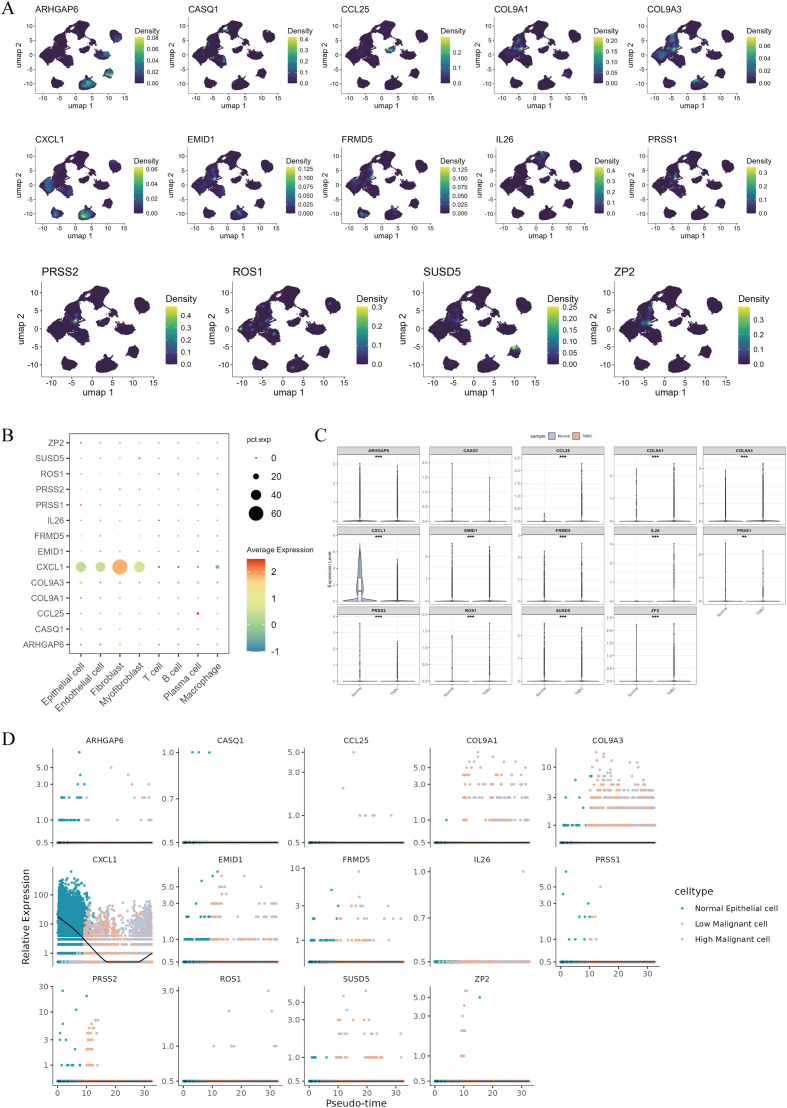
Expression patterns and dynamic changes of matrix - stiffness signature genes. **(A)**, Distribution of the expression of key MS signature genes in the UMAP-dimensionality-reduced single-cell space. **(B)**, Bubble plot of the expression levels of MS signature genes in each cell type. The shade of the color represents the average expression level, and the size of the circle represents the proportion of cells expressing the gene. **(C)**, Comparison of the expression differences of MS signature genes between TNBC tumor samples and normal breast samples at the tissue level. **(D)**, Diagram of the dynamic expression change patterns of MS signature genes along the pseudotemporal trajectory of epithelial cell differentiation.

To verify the biological functions of characteristic genes in TNBC development, a systematic analysis was performed on the 14 genes. As shown in **Figure 8A**, *ZP2, ROS1, PRSS1, and COL9A1* were specifically expressed in Epithelial cells. A comparison of signature gene expression between tumor and normal tissues revealed significant differential expression in 13 genes, suggesting a strong link between these prognostic markers and TNBC development (**Figure 8B**). We independently validated some of the genes (*ARHGAP6, CCL25, COL9A1, EMID1, FRMD5, SUSD5, and ZP2*) by RT-qPCR in clinical samples. The results showed that *ARHGAP6* was significantly downregulated in tumor tissues, while the remaining genes were significantly or markedly upregulated, consistent with the model-predicted results. This experimentally confirmed the gene-basis of the MS score model (**Supplementary Figure 3**).

Pseudotemporal analysis further revealed that during the malignant transformation of epithelial cells, the expressions of C*CL25, COL9A1, COL9A3, ROS1*, and *SUSD5* gradually increased, while the expressions of *CXCL1* and *PRSS2* gradually decreased (**Figure 8C**). In addition, correlation analysis showed that multiple signature genes (*ARHGAP6, COL9A1, COL9A3, CXCL1, EMID1, IL26, PRSS1, PRSS2, SUSD5, ZP2*) were significantly negatively correlated with the MS score (**Supplementary Figure 4**). *CXCL1* and *IL26* showed a positive correlation with STAT3 expression, whereas the MS score showed a negative correlation (**Supplementary Figure 5**). Collectively, these findings established the biological relevance of the prognostic model’s signature gene set in TNBC.

## 4 Discussion

TNBC is highly heterogeneous and invasive, and lacks effective targeted treatment options, resulting in generally poor prognosis for patients (33). MS in the tumor microenvironment serves as a critical physical regulator and plays a pivotal role in driving tumor progression (34). However, its heterogeneous characteristics and functional impacts at the single-cell level in TNBC remain unclear. Therefore, this study integrated single-cell transcriptome sequencing and large-scale bulk transcriptome data to systematically analyze the central role of MS-related genes in TNBC cell heterogeneity, the differentiation of functional states of malignant epithelial cells, and the remodeling of the immune microenvironment. Furthermore, based on the characteristics of MS-related genes, a robust prognostic prediction model was constructed and validated. This model not only has independent prognostic value but is also significantly associated with tumor immune characteristics and treatment response, providing a new multi-dimensional perspective and practical tool for risk stratification, mechanism exploration, and treatment strategy optimization in TNBC patients.

TNBC, which originates from epithelial cells, heavily relies on these cells to drive its malignant progression (35). TNBC cells exhibit a significant increase in the CNV score, which confirms their malignant nature. Notably, these malignant epithelial cells are not a homogeneous population. Instead, driven by the mechanical signals of MS, they show significant differentiation in functional heterogeneity. It is generally believed that the ERBB/MAPK signaling pathway and focal adhesion signals are activated when cells sense matrix stiffness, thereby promoting cell proliferation, survival, and migration (36, 37). However, this study found that in high-MS cells, the activities of these classical mechanotransduction-related pathways are instead inhibited. At the same time, stress-survival pathways such as FoxO, autophagy, and p53 are significantly enriched (38, 39). This phenomenon suggests that under continuous mechanical stimulation, tumor cells may reduce their dependence on the initial mechanotransduction pathways through a negative-feedback regulatory mechanism, and instead activate a more adaptable stress-survival network to cope with changes in the mechanical microenvironment.

Epithelial cells actively transmit signals to immune cells by specifically upregulating the ligand-receptor pairs of MIF-(CD74 + CXCR4/CD44) and MDK-NCL. MIF has been confirmed as a key immunosuppressive factor that can promote the expansion of regulatory T cells and the polarization of M2-type macrophages (40, 41). MDK, on the other hand, is involved in mediating the recruitment and activation of tumor-associated macrophages (42). This abnormal communication initiated by tumor cells and dominated by immunosuppressive molecules reveals a novel mechanism by which tumors actively shape the immunosuppressive microenvironment.

The prognostic model derived from MS-related genes is grounded in the ability of this gene signature to systematically reflect the mechanical microenvironment-driven malignant progression in TNBC. The MS-related DEGs are significantly enriched in classical mechanotransduction pathways such as ERK signal regulation, focal adhesion, and extracellular matrix-receptor interaction, indicating that MS may influence tumor biological behaviors by regulating cell-matrix interactions and downstream signaling networks (43, 44). Specifically, COL9A1/3 and EMID1 are involved in ECM remodeling and hardening; the down-regulation of ARHGAP6 is related to enhanced cytoskeletal response (45, 46). CCL25 and CXCL1/IL26 can regulate the immune microenvironment; and ROS1 can mediate intracellular pro-survival signals (47–49). These genes with diverse functions together form a complete pathological network, systematically linking biological processes such as matrix hardening, extracellular matrix remodeling, mechanotransduction, and immune microenvironment remodeling.

Notably, STAT3 is a key molecular hub linking chronic inflammation with tumorigenesis and development (50). As a core transcription factor of inflammatory signals in the tumor microenvironment, the persistent activation of STAT3 is one of the core mechanisms driving tumor malignant progression, immune escape, and treatment resistance. IL-26 can directly activate STAT3 (51). For instance, IL-6 activates the JAK-STAT3 pathway through its receptor, and the activated STAT3 can further transcriptionally upregulate the expression of genes, forming a powerful positive-feedback amplification loop that continuously maintains the inflammatory state of the tumor and promotes metastasis (52). In this study, CXCL1 and IL26, whose expression levels are positively correlated with STAT3, follow the classical inflammatory signaling pattern. However, the MS score is significantly negatively correlated with STAT3 activity. This finding further reveals that under the continuous drive of mechanical signals, tumor cells may undergo reprogramming of the core signaling network, shifting from a STAT3-dominated inflammation-dependent state to a non-inflammatory survival state centered on mechanical adaptation.

By developing a matrix stiffness-based prognostic model and systematically revealing its link to the immune microenvironment, this study nonetheless has certain limitations. The specific functional mechanisms of prognostic signature genes in matrix stiffness signal transduction remain unclear, and in-depth functional experiments are needed to analyze their biological roles. Meanwhile, the conclusions of this prognostic model and its association with immunotherapy urgently need to be further verified in prospective clinical studies.

## 5 Conclusion

By integrating single-cell RNA sequencing and transcriptomic analyses, this study systematically elucidated the crucial role of MS-related characteristics in TNBC progression. The scoring model constructed based on MS-related genes could not only effectively distinguish the functional states of malignant epithelial cells, but also the derived prognostic model demonstrated better predictive performance than traditional indicators in multiple cohorts. Moreover, it had the value of synergistic stratification with tumor mutational burden. This score also showed a dual potential for predicting treatment response. Patients with low scores showed greater sensitivity to both chemotherapy and targeted agents. In contrast, a high score was a significant predictor of resistance to immunotherapy. At the mechanistic level, signature genes such as *CCL25* and *COL9A1* were confirmed to be involved in the malignant progression of TNBC. Collectively, these findings suggest that matrix stiffness characteristics provide a new molecular perspective and theoretical basis for the prognostic assessment, risk stratification, and optimization of treatment strategies for TNBC.

## Data Availability

The original contributions presented in the study are included in the article/Supplementary Material. Further inquiries can be directed to the corresponding author.

## Supplementary material

Supplementary Figure 1 Molecular characteristics related to the immune microenvironment in high- and low-MS groups. **(A)**, Expression levels of tumor-associated antigens (TAA) and major histocompatibility complex (MHC) molecules in the high- and low-MSscore groups. **(B)**, Comparison of the expression of key cell-communication pathways between epithelial cells and other cell types in the high- and low-MS score groups.

Supplementary Figure 2 Association between MS score and immune infiltration and drug sensitivity. **(A)**, ssGSEA was used to evaluate the levels of immune cell infiltration in the high - and low-MS score groups, and a radar chart was used to display the correlation patterns between the scores of each immune cell and the MS score. **(B)**, Based on genomics data, the differences in the half-maximal inhibitory concentration (IC50) of common chemotherapeutic drugs and targeted drugs between the high- and low-MS score groups were predicted and compared.

Supplementary Figure 3 Detection of the mRNA expression levels of key MS signature genes (ARHGAP6, CCL25, COL9A1, EMID1, FRMD5, SUSD5, ZP2) in triple-negative breast cancer samples by RT-qPCR.

Supplementary Figure 4 Correlation analysis between the MS score and the expression of characteristic genes.

Supplementary Figure 5 Correlation analysis of the expression of characteristic genes, MS score, and STAT3 expression.
