# Supplementary figures and images for: Novel insights into triple-negative breast cancer heterogeneity, prognosis, and treatment response based on matrix stiffness: a combined single-Cell and transcriptome analysis
Source: Front Oncol. 2026 May 7;16:1821887. doi: 10.3389/fonc.2026.1821887 (PMC13189971; doi:10.3389/fonc.2026.1821887)

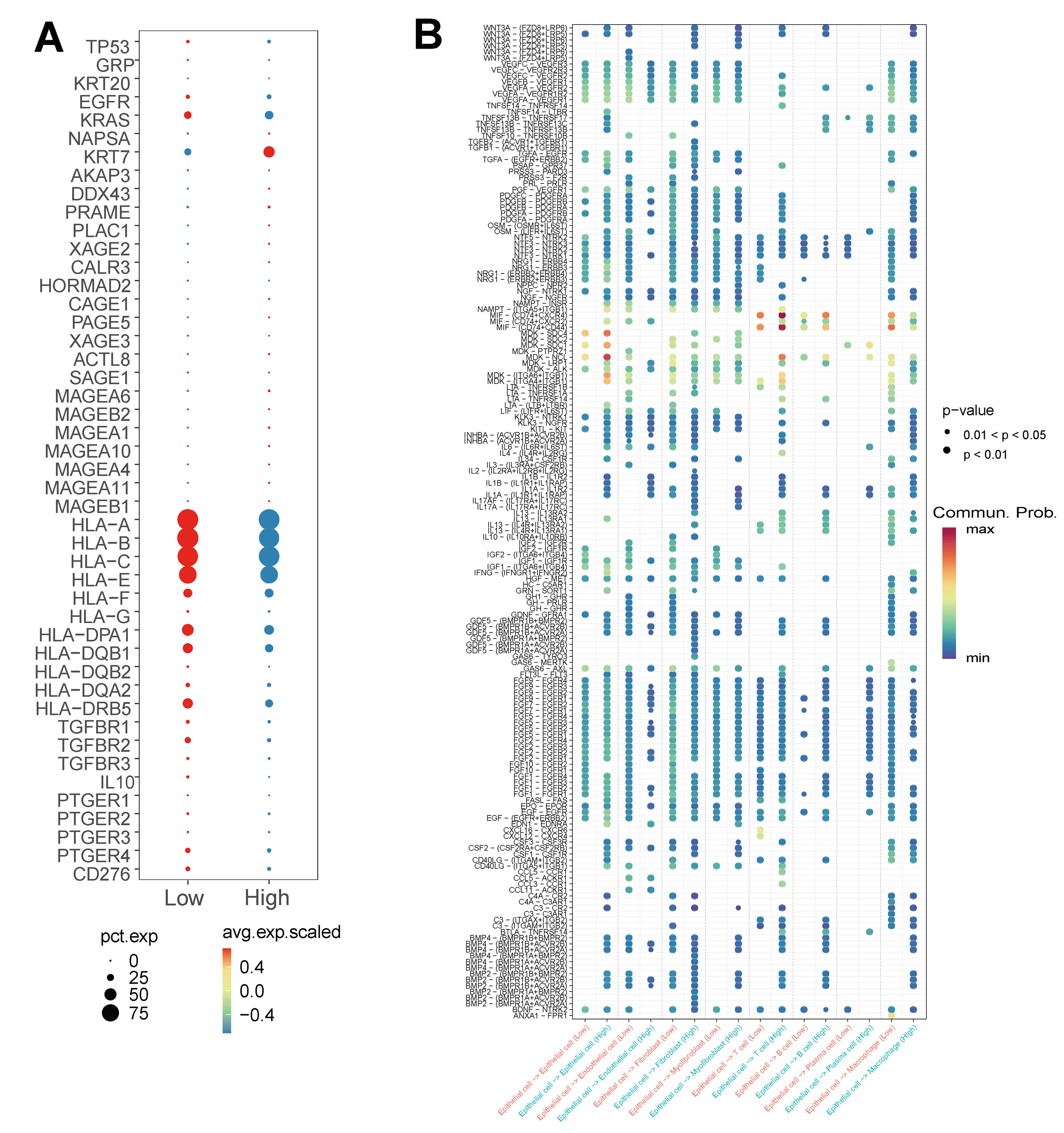

Supplement: Supplementary file 1 [file Image1.tif]

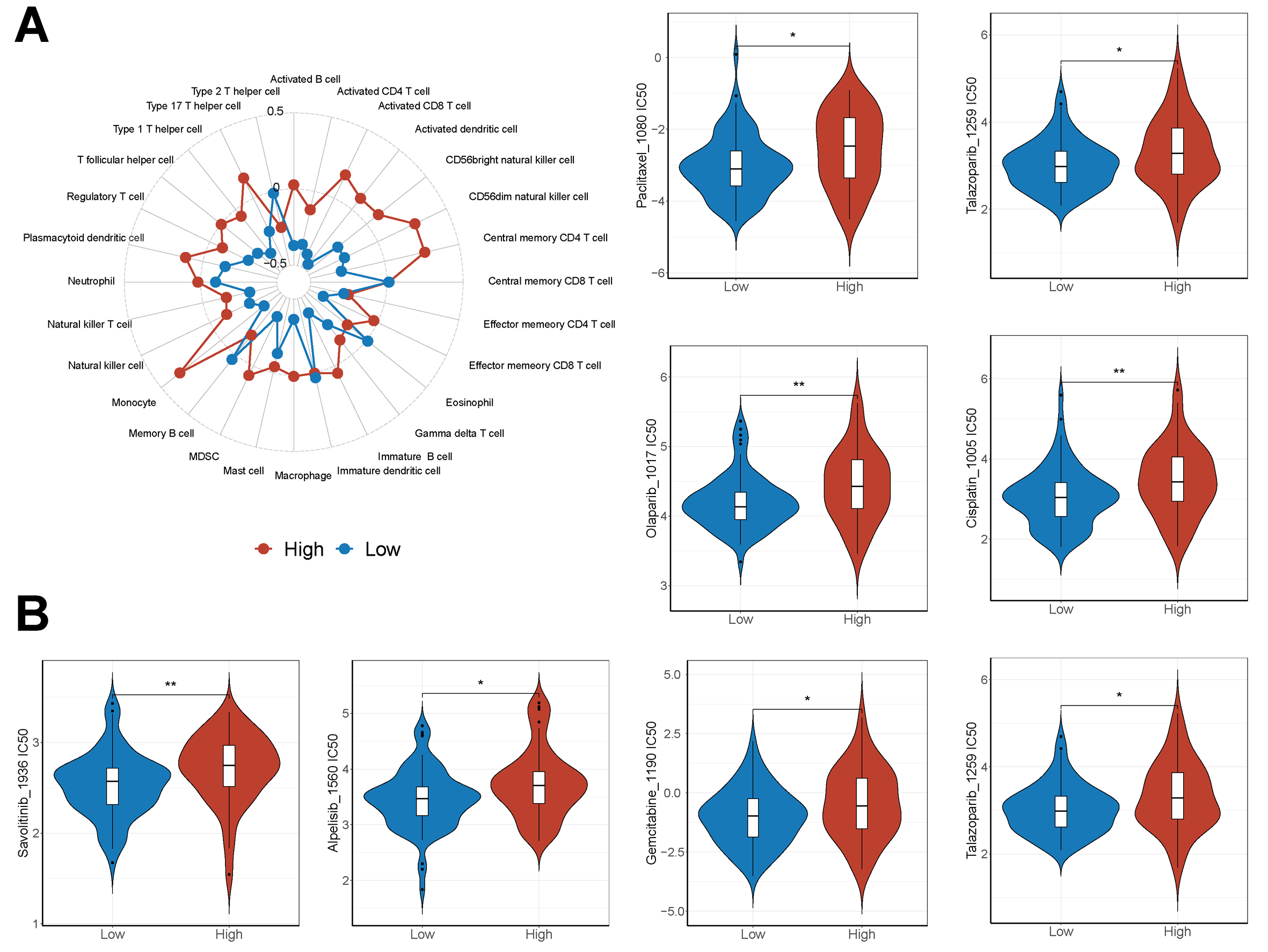

Supplement: Supplementary file 2 [file Image2.tif]

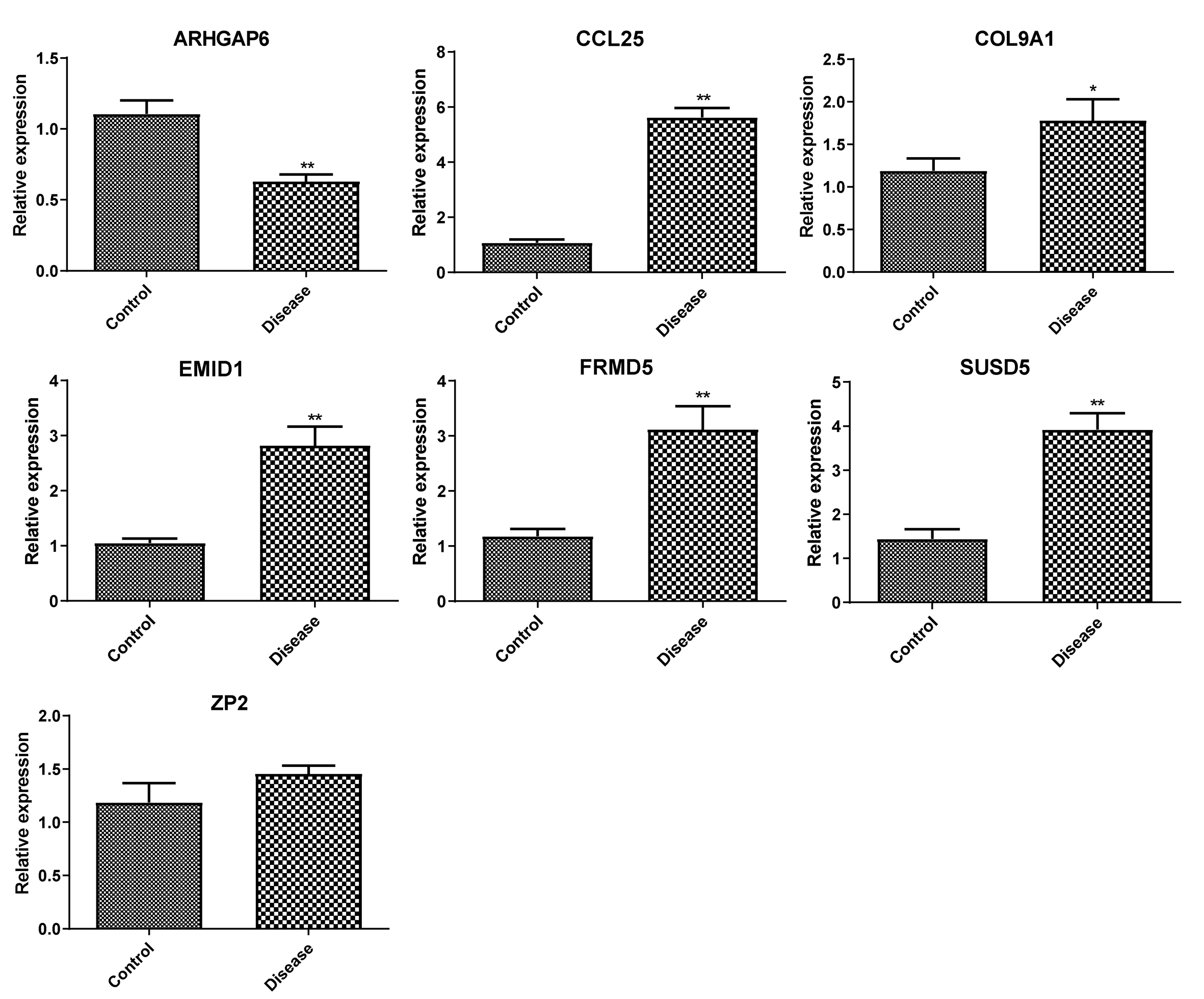

Supplement: Supplementary file 3 [file Image3.tif]

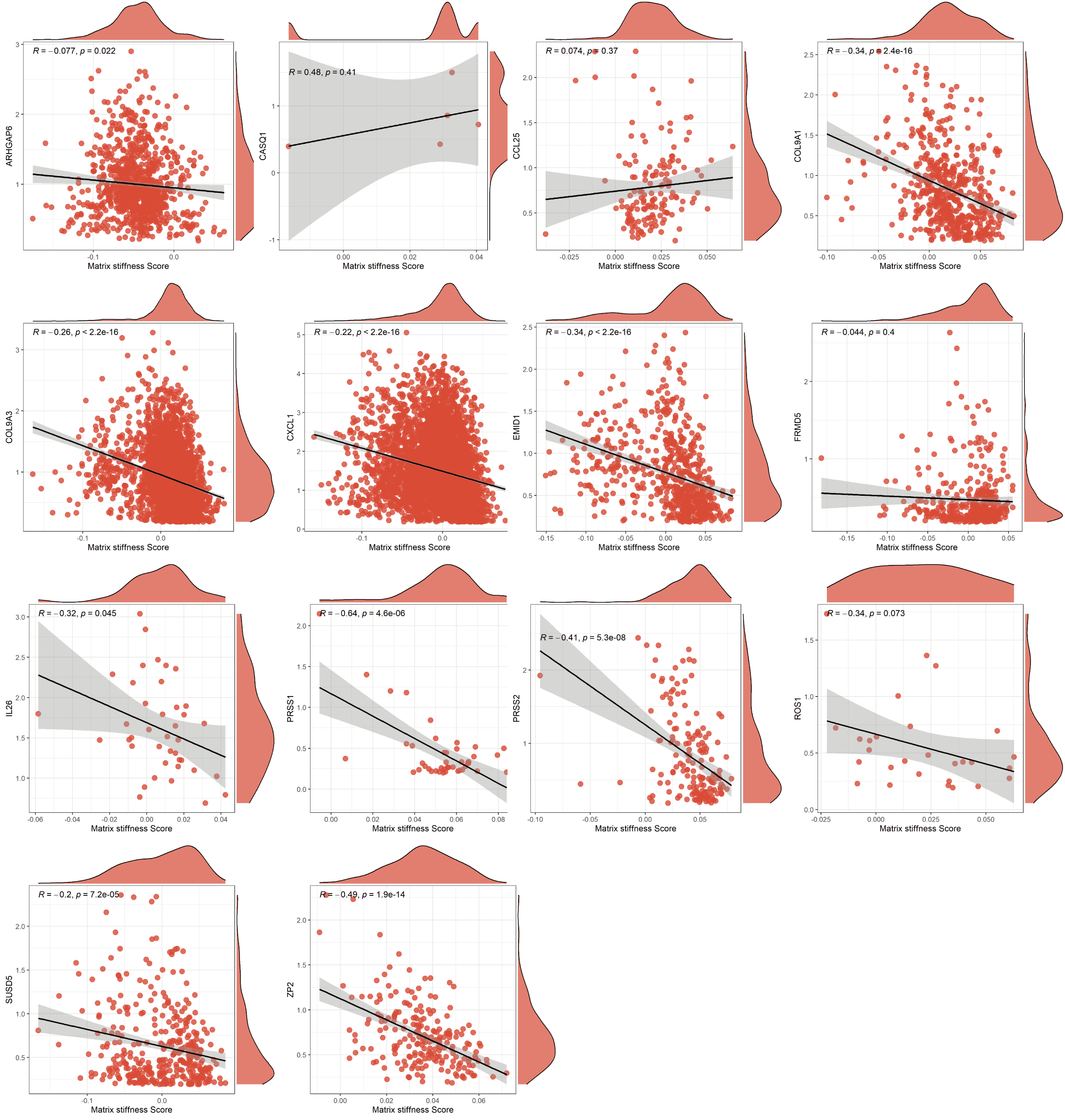

Supplement: Supplementary file 4 [file Image4.tiff]

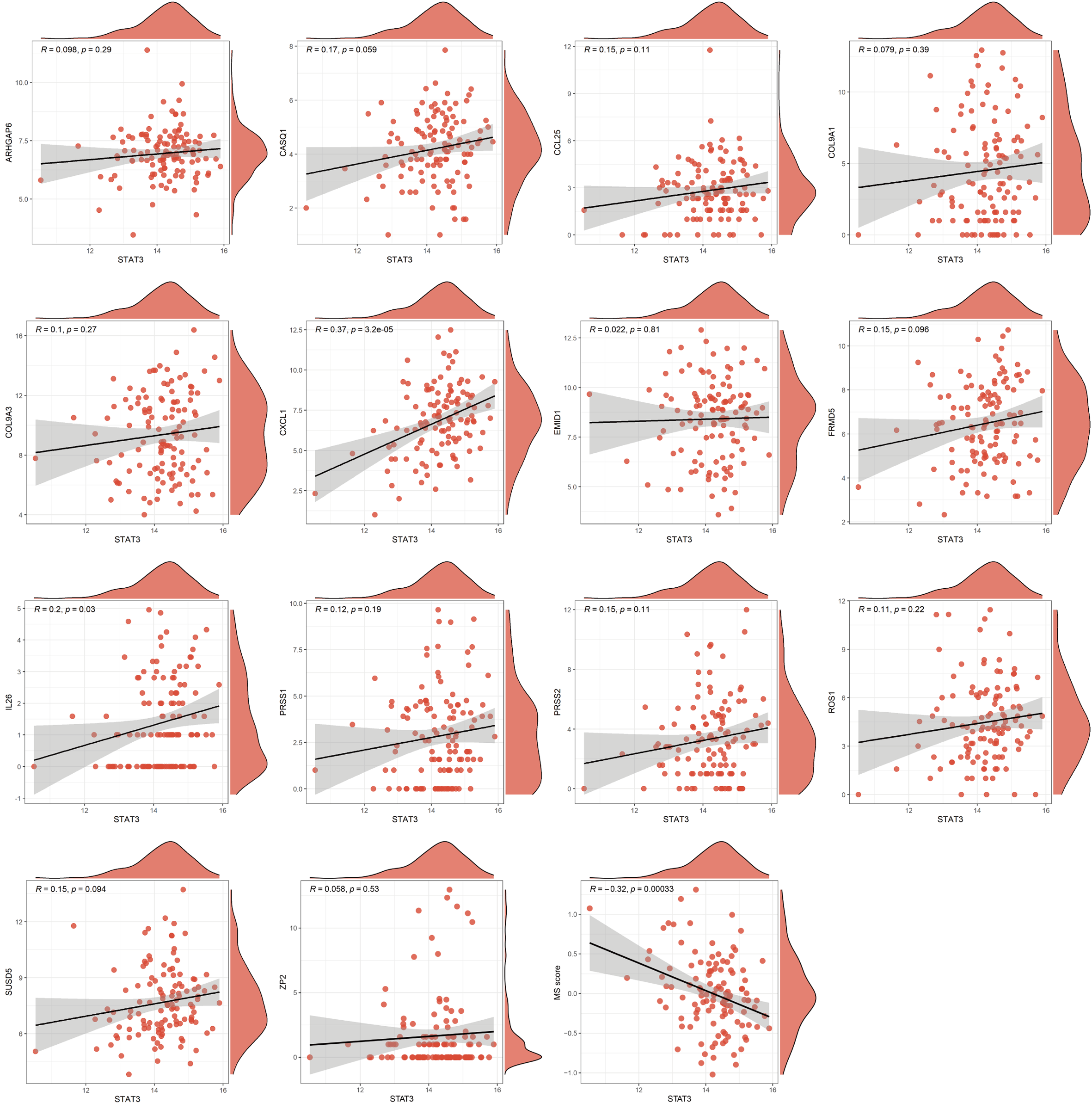

Supplement: Supplementary file 5 [file Image5.tif]
