# Supplementary material for: Novel insights into triple-negative breast cancer heterogeneity, prognosis, and treatment response based on matrix stiffness: a combined single-Cell and transcriptome analysis
Source: Front Oncol. 2026 May 7;16:1821887. doi: 10.3389/fonc.2026.1821887 (PMC13189971; doi:10.3389/fonc.2026.1821887)
Supplement: Supplementary Figure 1 — Molecular characteristics related to the immune microenvironment in high- and low-MS groups. (A), Expression levels of tumor-associated antigens (TAA) and major histocompatibility complex (MHC) molecules in the high- and low-MSscore groups. (B), Comparison of the expression of key cell-communication pathways between epithelial cells and other cell types in the high- and low-MS score groups. [file Table1.docx]

**Supplementary materials**

Table S1. Primer information

| Primer Name | Primer Sequence (5'–3') |
| --- | --- |
| GAPDH-hF | TGACAACTTTGGTATCGTGGAAGG |
| GAPDH-hR | AGGCAGGGATGATGTTCTGGAGAG |
| ARHGAP6-hF | CAGGACTTGCAGAGGGACGAG |
| ARHGAP6-hR | CATTCGGTGTTTCTGAGGTTGA |
| CCL25-hF | CTGCCTGCTGCGATATTCTAC |
| CCL25-hR | GTCTGCGTGTTGTGGTGGAG |
| COL9A1-hF | GAGCACCGACAGATCAGCAC |
| COL9A1-hR | CCCTCAAACCAAGAGCACCAG |
| EMID1-hF | GGGCGGACATGCAACCAACT |
| EMID1-hR | GCCCTTGCTCAGCCTCAGACAT |
| FRMD5-hF | CGCCAACAATACGGAGTTTC |
| FRMD5-hR | CCGCTGCTTATCTGGGTCTA |
| SUSD5-hF | CCGTGAATGCTTCCGAGACT |
| SUSD5-hR | TGGTTGCCATGATCGTTGAG |
| ZP2-hF | GGGAGAAGAGTAGGAGTGAAA |
| ZP2-hR | AGCCCATTTAGTGATTTGAC |
